# Supplementary material for: Genetic and Transcriptomic Characteristics of RhlR-Dependent Quorum Sensing in Cystic Fibrosis Isolates of Pseudomonas aeruginosa
Source: mSystems. 2022 Apr 11;7(2):e00113-22. doi: 10.1128/msystems.00113-22 (PMC9040856; doi:10.1128/msystems.00113-22)
Supplement: FIG S1 [file msystems.00113-22-s0001.pdf]

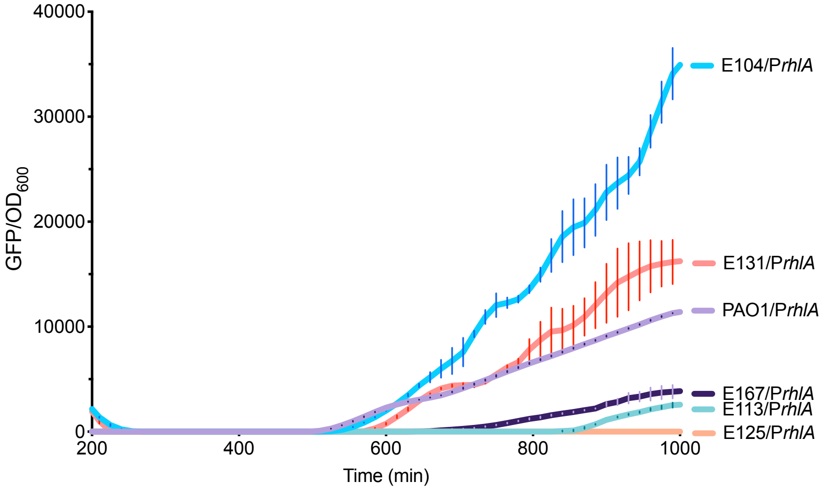

Figure S1. P$_{rhlA}$-*gfp* induction in a cohort of CF isolates. Total GFP fluorescence was monitored at 15 min intervals as described in the Materials and Methods. Values are normalized by cell density and corrected for background fluorescence using a promoterless *gfp* control plasmid. Data displayed are means of three biological replicates with standard error shown. Data were smoothed by reporting the mean of three consecutive measurements.
