# Supplementary material for: Genetic and Transcriptomic Characteristics of RhlR-Dependent Quorum Sensing in Cystic Fibrosis Isolates of Pseudomonas aeruginosa
Source: mSystems. 2022 Apr 11;7(2):e00113-22. doi: 10.1128/msystems.00113-22 (PMC9040856; doi:10.1128/msystems.00113-22)
Supplement: TABLE S1 [file msystems.00113-22-s0004.pdf]

Table S1. CF isolate genomic features.

Strain	Length (bp)	Features[1]	Plasmid (bp)
PAO1	6264403	5901	-
E104	6680448	6414	-
E113	6864839	6544	-
E125	6895810	6798	-
E131	6666693	6379	-
E167	6831875	6661	47445

[1]Features enumerated from RAST annotations (1).

References

1. Aziz RK, Bartels D, Best AA, DeJongh M, Disz T, Edwards RA, Formsma K, Gerdes S, Glass EM, Kubal M, Meyer F, Olsen GJ, Olson R, Osterman AL, Overbeek RA, McNeil LK, Paarmann D, Paczian T, Parrello B, Pusch GD, Reich C, Stevens R, Vassieva O, Vonstein V, Wilke A, Zagnitko O. 2008. The RAST Server: rapid annotations using subsystems technology. *BMC Genomics* 9:75.
