# Supplementary material for: Genetic and Transcriptomic Characteristics of RhlR-Dependent Quorum Sensing in Cystic Fibrosis Isolates of Pseudomonas aeruginosa
Source: mSystems. 2022 Apr 11;7(2):e00113-22. doi: 10.1128/msystems.00113-22 (PMC9040856; doi:10.1128/msystems.00113-22)
Supplement: TABLE S2 [file msystems.00113-22-s0005.pdf]

Table S2. PAO1 genes with nonsynonymous mutations in all CF isolates.

Locus tag[1]	Gene name[2]	Function[2]	Full deletion
PA0078	*tssL1*	TssL1	
PA0084	*tssC1*	TssC1	
PA0093	*tse6*	Tse6	
PA0170		hypothetical protein	
PA0225		probable transcriptional regulator	
PA0241		probable major facilitator superfamily (MFS) transporter	
PA0256		hypothetical protein	
PA0285		conserved hypothetical protein	
PA0287	*gpuP*	3-guanidinopropionate transport protein	
PA0373	*ftsY*	signal recognition particle receptor FtsY	
PA0457		hypothetical protein	
PA0548	*tktA*	transketolase	
PA0558		conserved hypothetical protein	
PA0559		conserved hypothetical protein	
PA0561		hypothetical protein	Yes
PA0715		hypothetical protein	Yes
PA0716		hypothetical protein	Yes
PA0717		hypothetical protein of bacteriophage Pf1	Yes
PA0724		probable coat protein A of bacteriophage Pf1	Yes
PA0729		hypothetical protein	Yes
PA0787		hypothetical protein	
PA0807	*ampDh3*	AmpDh3	
PA0842		probable glycosyl transferase	
PA0860		probable ATP-binding/permease fusion ABC transporter	Yes
PA0919		alanyl-phosphatidylglycerol hydrolase	Yes
PA0946		hypothetical protein	Yes
PA0948		hypothetical protein	
PA0977		hypothetical protein	Yes
PA0983		conserved hypothetical protein	Yes
PA0985	*pyoS5*	pyocin S5	Yes
PA1012		conserved hypothetical protein	
PA1014	*wapB*	1,2-glucosyltransferase WapB	
PA1019	*mucK*	cis,cis-muconate transporter MucK	
PA1128		probable transcriptional regulator	
PA1150	*pys2*	pyocin S2	
PA1218		hypothetical protein	
PA1219		hypothetical protein	
PA1229		probable transcriptional regulator	
PA1240		probable enoyl-CoA hydratase/isomerase	
PA1245	*aprX*	AprX	
PA1283		probable transcriptional regulator	
PA1286		probable major facilitator superfamily (MFS) transporter	
PA1356		hypothetical protein	
PA1368		hypothetical protein	Yes
PA1369		hypothetical protein	Yes
PA1370		hypothetical protein	Yes
PA1371		hypothetical protein	Yes
PA1372		hypothetical protein	Yes

PA1381		hypothetical protein	Yes
PA1382	*xqhB*	probable type II secretion system protein	Yes
PA1383		hypothetical protein	Yes
PA1384	*galE*	UDP-glucose 4-epimerase	Yes
PA1385		probable glycosyl transferase	Yes
PA1386		probable ATP-binding component of ABC transporter	Yes
PA1387		hypothetical protein	Yes
PA1388		hypothetical protein	Yes
PA1389		probable glycosyl transferase	Yes
PA1390		probable glycosyl transferase	Yes
PA1391		probable glycosyl transferase	Yes
PA1392		hypothetical protein	Yes
PA1393	*cysC*	adenosine 5'-phosphosulfate (APS) kinase	Yes
PA1416		conserved hypothetical protein	
PA1430	*lasR*	transcriptional regulator LasR	
PA1495		hypothetical protein	
PA1614	*gpsA*	glycerol-3-phosphate dehydrogenase, biosynthetic	
PA1620		hypothetical protein	
PA1621		probable hydrolase	
PA1714	*exsD*	ExsD	Yes
PA1721	*pscH*	type III export protein PscH	
PA1749		hypothetical protein	
PA1813		probable hydroxyacylglutathione hydrolase	
PA1842		hypothetical protein	
PA1887		hypothetical protein	Yes
PA1888		hypothetical protein	Yes
PA1892		hypothetical protein	
PA1909		hypothetical protein	
PA1914		conserved hypothetical protein	
PA1935		hypothetical protein	Yes
PA1939		hypothetical protein	Yes
PA2047	*cmrA*	probable transcriptional regulator	
PA2048		hypothetical protein	
PA2073		probable transporter (membrane subunit)	Yes
PA2074		hypothetical protein	Yes
PA2118a		hypothetical protein	
PA2139		hypothetical protein	Yes
PA2192		conserved hypothetical protein	
PA2218		hypothetical protein	Yes
PA2219	*opdE*	membrane protein OpdE	Yes
PA2220	*oprR*	probable transcriptional regulator	Yes
PA2229		conserved hypothetical protein	
PA2233	*pslC*	PslC	
PA2335		probable TonB-dependent receptor	
PA2336		hypothetical protein	
PA2340		probable binding-protein-dependent maltose/mannitol transport protein	Yes
PA2403	*fpvG*	FpvG	
PA2497		probable transcriptional regulator	
PA2618		hypothetical protein	
PA2626	*trmU*	tRNA methyltransferase	

PA2707		hypothetical protein	
PA2730		hypothetical protein	Yes
PA2731		Uncharacterized protein	Yes
PA2732		hypothetical protein	Yes
PA2733		conserved hypothetical protein	Yes
PA2734		hypothetical protein	Yes
PA2735		probable restriction-modification system protein	Yes
PA2736		hypothetical protein	Yes
PA2745		probable hydrolase	
PA2813		probable glutathione S-transferase	
PA2846		probable transcriptional regulator	
PA2870		diguanylate cyclase	
PA2885	*atuR*	putative repressor of atu genes	
PA2910		conserved hypothetical protein	
PA2922		probable hydrolase	
PA3146	*wbpK*	probable NAD-dependent epimerase/dehydratase WbpK	Yes
PA3147	*wbpJ*	probable glycosyl transferase WbpJ	Yes
PA3148	*wbpI*	UDP-N-acetylglucosamine 2-epimerase WbpI	Yes
PA3149	*wbpH*	probable glycosyltransferase WbpH	Yes
PA3150	*wbpG*	LPS biosynthesis protein WbpG	Yes
PA3151	*hisF2*	imidazoleglycerol-phosphate synthase, cyclase subunit	Yes
PA3152	*hisH2*	glutamine amidotransferase	Yes
PA3153	*wzx*	O-antigen translocase	Yes
PA3154	*wzy*	B-band O-antigen polymerase	Yes
PA3157		probable acetyltransferase	Yes
PA3163	*cmk*	cytidylate kinase	
PA3187		probable ATP-binding component of ABC transporter	
PA3285		probable sigma-70 factor, ECF subfamily	
PA3302		conserved hypothetical protein	
PA3364	*amiC*	aliphatic amidase expression-regulating protein	
PA3373		conserved hypothetical protein	
PA3374		conserved hypothetical protein	
PA3407	*hasAp*	heme acquisition protein HasAp	
PA3455		conserved hypothetical protein	
PA3492		conserved hypothetical protein	
PA3565		probable transcriptional regulator	Yes
PA3589		probable acyl-CoA thiolase	
PA3598		conserved hypothetical protein	
PA3670		hypothetical protein	
PA3719	*armR*	antirepressor for MexR, ArmR	Yes
PA3780		hypothetical protein	Yes
PA3790	*oprC*	Putative copper transport outer membrane porin OprC precursor	
PA3840		conserved hypothetical protein	
PA3843		hypothetical protein	Yes
PA3866		pyocin S4	Yes
PA3867		probable DNA invertase	Yes
PA3868		hypothetical protein	Yes
PA3869		hypothetical protein	Yes
PA3890	*opuCB*	OpuC ABC transporter, permease protein, OpuCB	
PA3893		conserved hypothetical protein	
PA3931		conserved hypothetical protein	

PA3932		probable transcriptional regulator	Yes
PA3933	*betT3*	BetT3	
PA3959		hypothetical protein	
PA4014		hypothetical protein	
PA4188		conserved hypothetical protein	
PA4192		probable ATP-binding component of ABC transporter	
PA4193		probable permease of ABC transporter	
PA4195		probable binding protein component of ABC transporter	
PA4211	*phzB1*	probable phenazine biosynthesis protein	
PA4313a		hypothetical protein	
PA4330		probable enoyl-CoA hydratase/isomerase	
PA4442	*cysN*	ATP sulfurylase GTP-binding subunit/APS kinase	
PA4466		probable phosphoryl carrier protein	
PA4525	*pilA*	type 4 fimbrial precursor PilA	Yes
PA4526	*pilB*	type 4 fimbrial biogenesis protein PilB	
PA4546	*pilS*	two-component sensor PilS	
PA4549	*fimT*	type 4 fimbrial biogenesis protein FimT	
PA4554	*pilY1*	type 4 fimbrial biogenesis protein PilY1	
PA4620		hypothetical protein	
PA4628	*lysP*	lysine-specific permease	
PA4642		hypothetical protein	
PA4664	*prmC*	S-adenosylmethionine-dependent methyltransferase, PrmC	
PA4714		conserved hypothetical protein	
PA4730	*panC*	pantoate--beta-alanine ligase	
PA4790		conserved hypothetical protein	
PA4797		probable transposase	Yes
PA4872		hypothetical protein	
PA4884		hypothetical protein	
PA4911		probable permease of ABC branched-chain amino acid transporter	
PA4914	*amaR*	transcriptional regulator, AmaR	
PA4916	*nrtR*	Nudix-related transcriptional regulator NrtR	
PA4988	*waaA*	3-deoxy-D-manno-octulosonic-acid (KDO) transferase	
PA5030		probable major facilitator superfamily (MFS) transporter	
PA5069	*tatB*	translocation protein TatB	
PA5082	*dguC*	DguC	
PA5085	*dguR*	DguR	
PA5112	*estA*	esterase EstA	
PA5122		hypothetical protein	
PA5141	*hisA*	phosphoribosylformimino-5-aminoimidazole carboxamide	Yes
PA5246		conserved hypothetical protein	
PA5263	*argH*	argininosuccinate lyase	Yes
PA5296	*rep*	ATP-dependent DNA helicase Rep	Yes
PA5345	*recG*	ATP-dependent DNA helicase RecG	
PA5349	*rubB*	rubredoxin reductase	
PA5382		probable transcriptional regulator	Yes
PA5418	*soxA*	sarcosine oxidase alpha subunit	
PA5500	*znuC*	zinc transport protein ZnuC	
PA5502		hypothetical protein	

[1]PAO1 locus tags as determined by sequence homology.

[2]Gene names and functions from Pseudomonas.com (1).

References

1. Winsor GL, Griffiths EJ, Lo R, Dhillon BK, Shay JA, Brinkman FS. 2016. Enhanced annotations and features for comparing thousands of *Pseudomonas* genomes in the *Pseudomonas* genome database. *Nucleic Acids Res* 44:D646-53.
