# Supplementary material for: Genetic and Transcriptomic Characteristics of RhlR-Dependent Quorum Sensing in Cystic Fibrosis Isolates of Pseudomonas aeruginosa
Source: mSystems. 2022 Apr 11;7(2):e00113-22. doi: 10.1128/msystems.00113-22 (PMC9040856; doi:10.1128/msystems.00113-22)
Supplement: TABLE S3 [file msystems.00113-22-s0006.pdf]

Table S3. Genes present in all isolates, but not present in PAO1.

Strain-specific gene ID[a]					Gene length (bp)	Gene cluster	Annotated function[a]
E104	E113	E125	E131	E167			
2913680624	2913676237	2870687275	2913687009	2870696693	100		hypothetical protein
2913680625	2913676417	2870685150	2913687010	2870696694	98		transposase
2913680836	2913674339	2870683918	2913687221	2870696905	402		Integrase
2913681549	2913675136	2870684674	2913687875	2870697559	319		nucleoside-diphosphate-sugar epimerase
2913679820	2913673343	2870682899	2913686204	2870695889	145		hypothetical protein
2913682201	2913676275	2870685342	2913688528	2870697914	1039		Cu(I)/Ag(I) efflux system membrane protein CusA/SilA
2913682210	2913676267	2870685351	2913688537	2870697923	691		Cu+-exporting ATPase
2913682302	2913677723	2870685390	2913688582	2870697968	464		integrating conjugative element protein (TIGR03755 family)
2913682303	2913677722	2870685391	2913688583	2870697969	316		integrating conjugative element protein (TIGR03756 family)
2913682326	2913677702	2870685414	2913688606	2870697992	730		conjugative coupling factor TraD (TOL family)
2913682337	2913677684	2870685420	2913688617	2870698003	759		SNF2 family DNA or RNA helicase
2913682748	2913676288	2870685965	2913689046	2870698819	180	GC-1	type VI protein secretion system component VasK
2913682749	2913676289	2870685966	2913689047	2870698820	399	GC-1	DNA-binding PucR family transcriptional regulato
2913682750	2913676290	2870685967	2913689048	2870698821	288	GC-1	ectoine hydroxylase-related dioxygenase (phytanoyl-CoA dioxygenase family)
2913682751	2913676291	2870685968	2913689049	2870698822	320	GC-1	photosystem II stability/assembly factor-like uncharacterized protein
2913682752	2913676292	2870685969	2913689050	2870698823	800	GC-1	predicted RND superfamily exporter protein
2913682753	2913676293	2870685970	2913689051	2870698824	548	GC-1	hypothetical protein
2913682754	2913676294	2870685971	2913689052	2870698825	474	GC-1	hypothetical protein
2913682755	2913676295	2870685972	2913689053	2870698826	220	GC-1	hypothetical protein
2913682756	2913676296	2870685973	2913689054	2870698827	455	GC-1	glycine/D-amino acid oxidase-like deaminating enzyme
2913682757	2913676297	2870685974	2913689055	2870698828	295	GC-1	ectoine hydroxylase-related dioxygenase (phytanoyl-CoA dioxygenase family
2913682758	2913676298	2870685975	2913689056	2870698829	193	GC-1	AcrR family transcriptional regulator
2913682759	2913676299	2870685976	2913689057	2870698830	508	GC-1	cation diffusion facilitator CzcD-associated flavoprotein CzcO
2913682760	2913676300	2870685977	2913689058	2870698831	291	GC-1	pimeloyl-ACP methyl ester carboxylesterase
2913682761	2913676301	2870685978	2913689059	2870698832	385	GC-1	alcohol dehydrogenase
2913682762	2913676302	2870685979	2913689060	2870698833	443	GC-1	amino acid transporter
2913682763	2913676303	2870685980	2913689061	2870698834	474	GC-1	aminobutyraldehyde dehydrogenase
2913682764	2913676304	2870685981	2913689062	2870698835	417	GC-1	diaminobutyrate-2-oxoglutarate transaminase
2913682765	2913676305	2870685982	2913689063	2870698836	319	GC-1	3-hydroxyisobutyrate dehydrogenase-like beta-hydroxyacid dehydrogenase
2913682766	2913676306	2870685983	2913689064	2870698837	50	GC-1	hypothetical protein
2913682767	2913676307	2870685984	2913689065	2870698838	552	GC-1	cation/acetate symporter
2913682768	2913676308	2870685985	2913689066	2870698839	107	GC-1	uncharacterized membrane protein (DUF485 family)
2913682769	2913676309	2870685986	2913689067	2870698840	549	GC-1	fatty-acyl-CoA synthase
2913682770	2913676310	2870685987	2913689068	2870698841	255	GC-1	NAD(P)-dependent dehydrogenase (short-subunit alcohol dehydrogenase family
2913682771	2913676311	2870685988	2913689069	2870698842	390	GC-1	acyl-CoA dehydrogenase
2913682772	2913676312	2870685989	2913689070	2870698843	118	GC-1	hypothetical protein

2913682773	2913676313	2870685990	2913689071	2870698844	351	GC-1	aminoglycoside phosphotransferase (APT) family kinase protein
2913682774	2913676314	2870685991	2913689072	2870698845	650	GC-1	propionate catabolism operon transcriptional regulator
2913682775	2913676315	2870685992	2913689073	2870698846	167	GC-1	hypothetical protein
2913682864	2913676395	2870686081	2913689161	2870698935	71		hypothetical protein
2913683112	2913676672	2870686330	2913689409	2870699183	291		4-hydroxy-tetrahydrodipicolinate synthase
2913683662	2913677091	2870686877	2913689959	2870699822	50		hypothetical protein
2913684055	2913677628	2870688001	2913690352	2870700216	288	GC-2	chromosome partitioning related protein ParA
2913684056	2913677629	2870688002	2913690353	2870700217	245	GC-2	hypothetical protein
2913684057	2913677630	2870688003	2913690354	2870700218	165	GC-2	hypothetical protein
2913684058	2913677631	2870688004	2913690355	2870700219	239	GC-2	hypothetical protein
2913684060	2913677632	2870688006	2913690357	2870700221	233	GC-2	hypothetical protein
2913684061	2913677635	2870688009	2913690358	2870700222	465	GC-2	replicative DNA helicase
2913684062	2913677636	2870688011	2913690359	2870700223	61	GC-2	formate dehydrogenase maturation protein FdhE
2913684063	2913677637	2870688012	2913690360	2870700224	175	GC-2	hypothetical protein
2913684064	2913677638	2870688013	2913690361	2870700225	85	GC-2	hypothetical protein
2913684065	2913677639	2870688014	2913690362	2870700226	79	GC-2	hypothetical protein
2913684066	2913677640	2870688015	2913690363	2870700227	77	GC-2	hypothetical protein
2913684068	2913677643	2870688017	2913690365	2870700229	89	GC-2	hypothetical protein
2913684069	2913677644	2870688018	2913690366	2870700230	576	GC-2	ParB family protein of integrating conjugative element (PFGI_1 class)
2913684070	2913677645	2870688019	2913690367	2870700231	255	GC-2	hypothetical protein
2913684071	2913677646	2870688020	2913690368	2870700232	441	GC-2	hypothetical protein
2913684075	2913677647	2870688023	2913690372	2870700236	242	GC-2	integrating conjugative element protein (TIGR03761 family)
2913684076	2913677648	2870688024	2913690373	2870700237	177	GC-2	hypothetical protein
2913684077	2913677650	2870688026	2913690374	2870700238	162	GC-2	single-strand DNA-binding protein
2913684079	2913677651	2870688033	2913690376	2870700240	639	GC-2	DNA topoisomerase-1
2913684081	2913677654	2870688035	2913690378	2870700242	629	GC-2	hypothetical protein
2913684082	2913677655	2870688036	2913690379	2870700243	657	GC-2	hypothetical protein
2913684083	2913677656	2870688039	2913690380	2870700244	374	GC-2	type IV pili sensor histidine kinase/response regulator
2913684084	2913677657	2870688040	2913690381	2870700245	569	GC-2	type IVB pilus formation R64 PilN family outer membrane protein
2913684085	2913677658	2870688041	2913690382	2870700246	441	GC-2	hypothetical protein
2913684086	2913677659	2870688042	2913690383	2870700247	177	GC-2	type IV pilus biogenesis protein PilP
2913684087	2913677660	2870688043	2913690384	2870700248	526	GC-2	type II secretory ATPase GspE/PulE/Tfp pilus assembly ATPase PilB-like protein
2913684088	2913677661	2870688044	2913690385	2870700249	359	GC-2	type II secretory pathway component PulF
2913684089	2913677662	2870688045	2913690386	2870700250	176	GC-2	type II secretory pathway pseudopilin PulG
2913684090	2913677663	2870688046	2913690387	2870700251	313	GC-2	twitching motility protein PilT
2913684091	2913677664	2870688047	2913690388	2870700252	442	GC-2	type II secretory pathway pseudopilin PulG
2913684092	2913677665	2870688048	2913690389	2870700253	145	GC-2	hypothetical protein
2913684094	2913677667	2870688050	2913690391	2870700255	129	GC-2	hypothetical protein

2913684095	2913677668	2870688051	2913690392	2870700256	58	GC-2	hypothetical protein
2913684098	2913677671	2870688054	2913690395	2870700259	162	GC-2	hypothetical protein
2913684100	2913677673	2870688056	2913690397	2870700261	65	GC-2	hypothetical protein
2913684102	2913677675	2870688057	2913690399	2870700263	235	GC-2	hypothetical protein
2913684104	2913677677	2870688059	2913690401	2870700265	116	GC-2	hypothetical protein
2913684105	2913677678	2870688060	2913690402	2870700266	256	GC-2	hypothetical protein
2913684106	2913677680	2870688061	2913690403	2870700267	120	GC-2	hypothetical protein
2913684107	2913677681	2870688062	2913690404	2870700268	84	GC-2	hypothetical protein
2913684108	2913677682	2870688063	2913690405	2870700269	201	GC-2	hypothetical protein
2913684109	2913677683	2870688064	2913690406	2870700270	395	GC-2	hypothetical protein
2913684117	2913676282	2870685438	2913690414	2870700278	116	GC-2	mercuric ion transport protein
2913684135	2913677696	2870688073	2913690432	2870700296	90	GC-2	hypothetical protein
2913684136	2913677697	2870688074	2913690433	2870700297	229	GC-2	soluble cytochrome b562
2913684137	2913677698	2870688075	2913690434	2870700298	251	GC-2	integrating conjugative element protein (TIGR03759 family
2913684138	2913677699	2870688076	2913690435	2870700299	193	GC-2	hypothetical protein
2913684139	2913677700	2870688077	2913690436	2870700300	166	GC-2	integrating conjugative element protein (TIGR03765 family)
2913684140	2913677701	2870688078	2913690437	2870700301	89	GC-2	hypothetical protein
2913684142	2913677703	2870688080	2913690439	2870700303	248	GC-2	integrating conjugative element membrane protein (TIGR03747 family)
2913684143	2913677705	2870688081	2913690440	2870700304	493	GC-2	superfamily I DNA/RNA helicase
2913684144	2913677706	2870688082	2913690441	2870700305	369	GC-2	3-dehydroquinate dehydratase
2913684145	2913677707	2870688083	2913690442	2870700306	99	GC-2	hypothetical protein
2913684146	2913677708	2870688084	2913690443	2870700307	97	GC-2	RAQPRD family integrative conjugative element protein
2913684147	2913677709	2870688085	2913690444	2870700308	49	GC-2	integrating conjugative element protein (TIGR03758 family)
2913684148	2913677710	2870688086	2913690445	2870700309	73	GC-2	integrating conjugative element membrane protein (TIGR03745 family)
2913684149	2913677711	2870688087	2913690446	2870700310	128	GC-2	conjugative transfer region protein (TIGR03750 family)
2913684150	2913677712	2870688088	2913690447	2870700311	219	GC-2	integrating conjugative element protein (TIGR03746 family)
2913684151	2913677713	2870688089	2913690448	2870700312	294	GC-2	integrating conjugative element protein (TIGR03749 family)
2913684152	2913677714	2870688090	2913690449	2870700313	501	GC-2	integrating conjugative element protein (TIGR03752 family)
2913684153	2913677715	2870688091	2913690450	2870700314	147	GC-2	conjugative transfer region lipoprotein (TIGR03751 family)
2913684154	2913677716	2870688092	2913690451	2870700315	980	GC-2	conjugative transfer ATPase
2913684155	2913677717	2870688093	2913690452	2870700316	94	GC-2	hypothetical protein
2913684159	2913677721	2870688099	2913690456	2870700320	63	GC-2	integrating conjugative element protein (TIGR03757 family)
2913684162	2913677724	2870687290	2913690459	2870700323	115	GC-2	Kef-type K+ transport system membrane component KefB
2913684163	2913677725	2870687291	2913690460	2870700324	511	GC-2	hypothetical protein
2913684166	2913677727	2870687293	2913690463	2870700327	90	GC-2	antitoxin ParD1/3/4
2913684167	2913677728	2870687294	2913690464	2870700328	116	GC-2	toxin ParE1/3/4
2913684173	2913677734	2870687295	2913690470	2870700334	639	GC-2	integrating conjugative element relaxase (TIGR03760 family)
2913684174	2913677735	2870687296	2913690471	2870700335	426	GC-2	Integrase

[a]Gene IDs and annotated functions from IMG/MER database (1, 2).

References

1. Chen I-MA, Chu K, Palaniappan K, Pillay M, Ratner A, Huang J, Huntemann M, Varghese N, White JR, Seshadri R, Smirnova T, Kirton E, Jungbluth SP, Woyke T, Eloe-Fadrosh EA, Ivanova NN, Kyrpides NC. 2018. IMG/M v.5.0: an integrated data management and comparative analysis system for microbial genomes and microbiomes. *Nucleic Acids Res* 47:D666-D677.
2. Mukherjee S, Stamatis D, Bertsch J, Ovchinnikova G, Sundaramurthi Jagadish C, Lee J, Kandimalla M, Chen I-MA, Kyrpides NC, Reddy TBK. 2020. Genomes OnLine Database (GOLD) v.8: overview and updates. *Nucleic Acids Res* 49:D723-D733.
