# Supplementary material for: Genetic and Transcriptomic Characteristics of RhlR-Dependent Quorum Sensing in Cystic Fibrosis Isolates of Pseudomonas aeruginosa
Source: mSystems. 2022 Apr 11;7(2):e00113-22. doi: 10.1128/msystems.00113-22 (PMC9040856; doi:10.1128/msystems.00113-22)
Supplement: TABLE S4 [file msystems.00113-22-s0007.pdf]

Table S4. RhlR panregulon.

					Fold-change[b]									
					E104		E113		E125		E131		167	
		Locus Tag[a]	Gene name[a]	Product Name[a]	FC	FCSE	FC	FCSE	FC	FCSE	FC	FCSE	FC	FCSE
		PA0050		hypothetical protein			3.36	1.23			2.39	1.27		
		PA0051	*phzH*	potential phenazine-modifying enzyme			3.22	1.24			4.25	1.21		
●		PA0052		hypothetical protein			2.01	1.22			2.99	1.17		
		PA0111		hypothetical protein									2.42	1.24
●	○	PA0122	*rahU*	rahU	5.03	1.14	48.53	1.33			26.13	1.33	6.47	1.11
		PA0123		probable transcriptional regulator			2.80	1.18						
		PA0130	*bauC*	3-Oxopropanoate dehydrogenase			2.18	1.24						
		PA0132	*bauA*	Beta-alanine:pyruvate transaminase			2.71	1.31						
		PA0195	*pntAA*	putative NAD(P) transhydrogenase, subunit alpha part 1			2.04	1.17						
		PA0195.1	*pntAB*	putative NAD(P) transhydrogenase, subunit alpha part 2			2.56	1.17						
		PA0197	*tonB2*	TonB2					2.19	1.23				
		PA0198	*exbB1*	transport protein ExbB			2.31	1.21						
		PA0199	*exbD1*	transport protein ExbD			3.34	1.22						
		PA0200		hypothetical protein			4.80	1.24						
		PA0208	*mdcA*	malonate decarboxylase alpha subunit			3.95	1.35						
		PA0209		conserved hypothetical protein			4.11	1.48						
		PA0210	*mdcC*	malonate decarboxylase delta subunit			6.97	1.46						
		PA0211	*mdcD*	malonate decarboxylase beta subunit			6.31	1.79						
		PA0213		hypothetical protein			3.46	1.46						
		PA0214		probable acyl transferase			2.78	1.32						
		PA0511	*nirJ*	heme d1 biosynthesis protein NirJ									2.63	1.23
		PA0534	*pauB1*	FAD-dependent oxidoreductase									2.81	1.15
		PA0546	*metK*	methionine adenosyltransferase			2.04	1.17						
		PA0547		probable transcriptional regulator			2.46	1.11						
●		PA0852	*cbpD*	chitin-binding protein CbpD precursor	2.01	1.12	8.73	1.24			8.93	1.11	2.52	1.11
		PA0865	*hpd*	4-hydroxyphenylpyruvate dioxygenase									5.02	1.56
		PA0866	*aroP2*	aromatic amino acid transport protein AroP2									4.71	1.23
		PA0870	*phhC*	aromatic amino acid aminotransferase									2.83	1.36
		PA0871	*phhB*	pterin-4-alpha-carbinolamine dehydratase									3.19	1.35
●	○	PA0997	*pqsB*	PqsB					2.31	1.12				
●	○	PA0998	*pqsC*	PqsC									2.04	1.25
	○	PA0999	*pqsD*	3-oxoacyl-[acyl-carrier-protein] synthase III									2.22	1.19
●	○	PA1000	*pqsE*	Quinolone signal response protein									2.64	1.13
●	○	PA1130	*rhlC*	rhamnosyltransferase 2			2.85	1.16			2.47	1.13		
●	○	PA1131		probable major facilitator superfamily (MFS) transporter			3.72	1.20						
		PA1168		hypothetical protein			5.01	1.44						
		PA1212		probable major facilitator superfamily (MFS) transporter			4.31	1.16			2.53	1.25		
		PA1213		hypothetical protein			5.97	1.17			3.32	1.34		
		PA1214		hypothetical protein	2.40	1.29	12.32	1.23			4.15	1.30		
		PA1215		hypothetical protein	5.23	1.16	8.83	1.15			4.73	1.32		
●		PA1216		hypothetical protein	7.75	1.14	36.49	1.19			12.30	1.47	2.91	1.13
●		PA1217		probable 2-isopropylmalate synthase			3.32	1.15						
●		PA1218		hypothetical protein			10.85	1.15			4.50	1.36		
		PA1219		hypothetical protein			18.84	1.21			3.72	1.32	2.46	1.32
		PA1220		hypothetical protein	3.23	1.30	19.64	1.19			3.61	1.33	3.45	1.24
●		**PA1221**		**hypothetical protein**	**5.19**	**1.20**	**20.07**	**1.16**	**2.71**	**1.19**	**4.03**	**1.27**	**5.78**	**1.14**
●	○	PA1245	*aprX*	AprX	2.21	1.09	3.68	1.27			5.32	1.23		
●	○	PA1246	*aprD*	alkaline protease secretion protein AprD			3.31	1.24			4.51	1.21		
●	○	PA1247	*aprE*	alkaline protease secretion protein AprE			2.90	1.20			6.27	1.27		

●	○	PA1248	*aprF*	Alkaline protease secretion outer membrane protein AprF precursor			2.36	1.23			4.18	1.25		
●		PA1249	*aprA*	alkaline metalloproteinase precursor			2.48	1.27			5.02	1.21		
●		PA1250	*aprI*	alkaline proteinase inhibitor AprI			2.23	1.19			3.31	1.23		
		PA1317	*cyoA*	cytochrome o ubiquinol oxidase subunit II			2.42	1.17						
		PA1319	*cyoC*	cytochrome o ubiquinol oxidase subunit III			2.18	1.24						
●	○	PA1431	*rsaL*	regulatory protein RsaL							2.52	1.26		
●	○	PA1432	*lasI*	autoinducer synthesis protein LasI							2.28	1.19	2.52	1.15
		PA1546	*hemN*	oxygen-independent coproporphyrinogen III oxidase			2.31	1.27						
		PA1550		hypothetical protein			2.64	1.17						
		PA1551		probable ferredoxin			2.47	1.12						
		PA1556	*ccoO2*	Cytochrome c oxidase, cbb3-type, CcoO subunit			2.59	1.26						
		PA1557	*ccoN2*	Cytochrome c oxidase, cbb3-type, CcoN subunit			3.25	1.15						
●	○	PA1656	*hsiA2*	HsiA2	4.73	1.10	18.47	1.15	2.05	1.12			4.27	1.13
●	○	PA1657	*hsiB2*	HsiB2	2.57	1.15	9.74	1.15					4.51	1.22
●	○	PA1658	*hsiC2*	HsiC2			4.83	1.19					2.56	1.13
	○	PA1659	*hsiF2*	HsiF2	2.33	1.25	5.22	1.20					2.69	1.19
●	○	PA1660	*hsiG2*	HsiG2			3.42	1.13						
●	○	PA1661	*hsiH2*	HsiH2			4.80	1.16						
	○	PA1662	*clpV2*	clpV2			2.93	1.12						
●	○	PA1663	*sfa2*	Sfa2			4.36	1.16					2.09	1.21
	○	PA1664	*orfX*	OrfX			5.32	1.20			4.91	1.60		
●	○	PA1665	*fha2*	Fha2			5.15	1.19					2.26	1.17
	○	PA1666	*lip2*	Lip2			3.60	1.23						
	○	PA1668	*dotU2*	DotU2			2.21	1.14						
	○	PA1670	*stp1*	Stp1			2.38	1.19					2.04	1.15
●		PA1784		hypothetical protein			2.04	1.27			2.00	1.14		
●	○	**PA1869**		**probable acyl carrier protein**	**28.72**	**1.14**	**79.34**	**1.20**	**5.78**	**1.13**	**14.77**	**1.21**	**10.05**	**1.16**
		PA1870		hypothetical protein			2.15	1.21						
●		PA1871	*lasA*	LasA protease precursor	2.48	1.13	13.94	1.21			5.85	1.14	2.25	1.10
●		PA1899	*phzA2*	probable phenazine biosynthesis protein	6.15	1.25	101.96	1.30			9.14	1.19	4.63	1.26
●		PA1900	*phzB2*	probable phenazine biosynthesis protein	4.60	1.18	49.57	1.31			12.33	1.16	2.64	1.15
		PA1901	*phzC2*	phenazine biosynthesis protein PhzC	4.62	1.38	130.13	1.20			9.22	1.23	2.46	1.21
		PA1902	*phzD2*	phenazine biosynthesis protein PhzD							13.88	1.26		
		PA1903	*phzE2*	phenazine biosynthesis protein PhzE			26.88	2.57					3.30	1.35
		PA1904	*phzF2*	probable phenazine biosynthesis protein			12.09	1.33						
		PA1905	*phzG2*	probable pyridoxamine 5'-phosphate oxidase			12.07	1.33			12.25	1.19		
		PA1920	*nrdD*	class III (anaerobic) ribonucleoside-triphosphate reductase subunit, NrdD			2.74	1.26						
		PA1979	*eraS*	sensor kinase, EraS			2.77	1.23						
●		PA2030		hypothetical protein									2.10	1.12
		PA2031		hypothetical protein							2.14	1.20	2.29	1.12
●		PA2066		hypothetical protein			2.34	1.24			3.92	1.25		
		PA2067		probable hydrolase			3.52	1.24			5.91	1.29		
●		PA2068		probable major facilitator superfamily (MFS) transporter			2.47	1.21			4.81	1.20		
●		PA2069		probable carbamoyl transferase			12.90	1.31			8.42	1.14		
●		PA2146		conserved hypothetical protein			2.26	1.29			4.14	1.38		
●		PA2159		conserved hypothetical protein							2.13	1.25		
●	○	**PA2193**	***hcnA***	**hydrogen cyanide synthase HcnA**	**23.85**	**1.29**	**65.55**	**1.22**	**9.66**	**1.25**	**8.91**	**1.30**	**39.16**	**1.28**
●	○	**PA2194**	***hcnB***	**hydrogen cyanide synthase HcnB**	**8.19**	**1.21**	**8.61**	**1.15**	**2.35**	**1.15**	**2.92**	**1.20**	**6.93**	**1.20**
●	○	**PA2195**	***hcnC***	**hydrogen cyanide synthase HcnC**	**3.43**	**1.13**	**8.52**	**1.13**	**2.06**	**1.12**	**2.74**	**1.22**	**5.53**	**1.10**
		PA2273	*soxR*	SoxR			2.31	1.10						
●		PA2274		hypothetical protein			34.03	1.26			12.96	1.17		
		PA2275		probable alcohol dehydrogenase (Zn-dependent)			8.87	1.27			3.91	1.19		
●		PA2300	*chiC*	chitinase			8.04	1.25			9.08	1.19		
●	○	PA2302	*ambE*	AmbE			2.91	1.36			3.53	1.18		

●	○	PA2303	*ambD*	AmbD			4.37	1.36			4.15	1.17		
●	○	PA2304	*ambC*	AmbC			3.43	1.33			3.74	1.20		
●	○	PA2305	*ambB*	AmbB			2.88	1.27			4.65	1.20		
		PA2321		gluconokinase					2.04	1.20				
		PA2327		probable permease of ABC transporter			2.37	1.22						
●		PA2328		hypothetical protein			3.78	1.26						
●		PA2329		probable ATP-binding component of ABC transporter			3.74	1.25						
●		PA2330		hypothetical protein			3.28	1.21						
●		PA2331		hypothetical protein			5.94	1.25						
		PA2507	*catA*	catechol 1,2-dioxygenase									5.89	1.46
		PA2508	*catC*	muconolactone delta-isomerase									5.21	1.42
		PA2509	*catB*	muconate cycloisomerase I									4.52	1.43
●		PA2564		hypothetical protein							2.57	1.09		
		PA2565		hypothetical protein							2.22	1.14		
●		PA2566		conserved hypothetical protein							2.37	1.17		
●	○	PA2570	*lecA*	LecA			16.50	1.25			2.03	1.21		
●	○	PA2587	*pqsH*	probable FAD-dependent monooxygenase			4.39	1.29			2.72	1.12		
●		PA2588		probable transcriptional regulator			4.37	1.14			3.36	1.13		
●		PA2589		hypothetical protein			2.26	1.22						
		PA2590		hypothetical protein	2.03	1.15								
●	○	**PA2591**	***vqsR***	**VqsR**	**13.63**	**1.12**	**10.87**	**1.32**	**6.89**	**1.10**	**3.34**	**1.21**	**14.16**	**1.08**
●		PA2592		probable periplasmic spermidine/putrescine-binding protein	4.76	1.11	10.74	1.18	2.28	1.10			3.82	1.06
		PA2593	*qteE*	quorum threshold expression element, QteE	2.03	1.20	12.76	1.17					2.03	1.13
		PA2594		conserved hypothetical protein			2.81	1.14						
		PA2788		probable chemotaxis transducer			2.44	1.18						
●		PA3022		hypothetical protein			2.23	1.10						
●	○	PA3104	*xcpP*	secretion protein XcpP			2.16	1.12						
		PA3318		hypothetical protein							2.17	1.24		
●		PA3325		conserved hypothetical protein	2.80	1.13	2.30	1.13						
●	○	**PA3326**	***azeA/clpP2***	**ClpP2**	**16.10**	**1.09**	**19.85**	**1.22**	**2.18**	**1.15**	**2.85**	**1.19**	**13.51**	**1.07**
●	○	**PA3327**	*azeB*	**probable non-ribosomal peptide synthetase**	**21.13**	**1.13**	**102.17**	**1.10**	**2.83**	**1.15**	**5.12**	**1.30**	**36.02**	**1.07**
	○	**PA3328**	*azeC*	**probable FAD-dependent monooxygenase**	**67.86**	**1.25**	**271.93**	**1.21**	**5.28**	**1.25**	**12.05**	**1.43**	**152.08**	**1.16**
●	○	**PA3329**	*azeD*	**hypothetical protein**	**18.71**	**1.17**	**83.98**	**1.19**	**3.03**	**1.26**	**8.22**	**1.44**	**45.88**	**1.18**
	○	**PA3330**	*azeE*	**probable short chain dehydrogenase**	**30.19**	**1.24**	**148.95**	**1.25**	**4.06**	**1.26**	**8.44**	**1.47**	**73.99**	**1.26**
●	○	PA3331		cytochrome P450	3.41	1.14	45.99	1.25			3.63	1.36	21.25	1.16
●	○	**PA3332**	*azeG*	**conserved hypothetical protein**	**38.93**	**1.26**	**98.62**	**1.33**	**3.96**	**1.37**	**18.39**	**1.54**	**70.46**	**1.27**
●	○	**PA3333**	***azeH/fabH2***	**3-oxoacyl-[acyl-carrier-protein] synthase III**	**22.65**	**1.17**	**73.33**	**1.30**	**2.15**	**1.21**	**14.31**	**1.48**	**40.57**	**1.16**
	○	**PA3334**		**probable acyl carrier protein**	**20.35**	**1.16**	**103.48**	**1.35**	**3.10**	**1.20**	**17.32**	**1.88**	**36.68**	**1.13**
	○	PA3335		hypothetical protein	13.42	1.14	26.91	1.32			4.02	1.39	19.14	1.17
	○	PA3336		probable major facilitator superfamily (MFS) transporter	4.74	1.19	11.19	1.31			2.85	1.36	7.96	1.30
●	○	PA3361	*lecB*	fucose-binding lectin PA-IIL			26.43	1.23			9.48	1.44	2.55	1.13
		PA3395	*nosY*	NosY protein							2.24	1.22		
		PA3397	*fprA*	FprA			2.92	1.13						
		PA3415		probable dihydrolipoamide acetyltransferase									2.17	1.23
		PA3441		probable molybdopterin-binding protein			2.06	1.20						
		PA3475	*pheC*	cyclohexadienyl dehydratase precursor			3.49	1.13			2.26	1.16		
●	○	**PA3476**	***rhlI***	**autoinducer synthesis protein RhlI**	**58.66**	**1.09**	**33.05**	**1.69**	**19.30**	**1.10**	**17.59**	**1.22**	**47.91**	**1.10**
●	○	PA3478	*rhlB*	rhamnosyltransferase chain B	2.18	1.11	10.40	1.23			5.03	1.16	2.26	1.16
●	○	**PA3479**	***rhlA***	**rhamnosyltransferase chain A**	**18.19**	**1.17**	**84.86**	**1.24**	**6.79**	**1.12**	**20.95**	**1.11**	**13.62**	**1.17**
		PA3519		hypothetical protein			2.17	1.26						
●		PA3520		hypothetical protein			4.24	1.19			2.34	1.16		
		PA3677	*mexJ*	MexJ			2.10	1.10						
		PA3718		probable major facilitator superfamily (MFS) transporter			3.06	1.17						
		PA3719	*armR*	antirepressor for MexR, ArmR			2.28	1.23						

		PA3720		hypothetical protein			2.05	1.24						
●	○	**PA3724**	***lasB***	**elastase LasB**	**11.07**	**1.10**	**103.15**	**1.36**	**3.31**	**1.11**	**25.49**	**1.17**	**15.78**	**1.12**
		PA3734		hypothetical protein			5.64	1.17			4.38	1.13		
		PA4067	*oprG*	Outer membrane protein OprG precursor			2.27	1.21						
●		PA4078		probable nonribosomal peptide synthetase			2.10	1.16			2.51	1.18		
		PA4127	*hpcG*	2-oxo-hept-3-ene-1,7-dioate hydratase									2.08	1.17
●		PA4128		conserved hypothetical protein			9.01	1.18					11.22	1.24
●		PA4129		hypothetical protein			57.11	1.13			4.46	1.36	29.28	1.20
●		PA4130		probable sulfite or nitrite reductase	2.17	1.11	38.90	1.10			4.92	1.16	16.16	1.14
●		PA4131		probable iron-sulfur protein	2.68	1.12	32.80	1.12			2.77	1.13	15.48	1.12
●		PA4132		conserved hypothetical protein			8.51	1.12					3.64	1.16
●		PA4133		cytochrome c oxidase subunit (cbb3-type)			44.37	1.16					6.15	1.15
●		PA4134		hypothetical protein			20.29	1.16					2.36	1.26
		PA4136		probable major facilitator superfamily (MFS) transporter			2.21	1.14						
●		**PA4141**		**hypothetical protein**	**3.80**	**1.07**	**23.59**	**1.16**	**2.69**	**1.08**	**63.25**	**1.38**	**4.67**	**1.16**
●		PA4142		probable secretion protein			5.19	1.17			2.15	1.12		
		PA4143		probable toxin transporter			3.10	1.12						
		PA4144		probable outer membrane protein precursor			3.33	1.15			2.44	1.22		
●		PA4205	*mexG*	hypothetical protein			43.50	1.21			6.82	1.18		
●		PA4206	*mexH*	probable Resistance-Nodulation-Cell Division (RND) efflux membrane fusion protein precursor			50.69	1.25			12.01	1.17		
●		PA4207	*mexI*	probable Resistance-Nodulation-Cell Division (RND) efflux transporter			36.89	1.23			10.22	1.18		
●		PA4208	*opmD*	probable outer membrane protein precursor			65.85	1.27			19.36	1.19		
●		PA4209	*phzM*	probable phenazine-specific methyltransferase			42.54	1.14			4.11	1.34	2.93	1.15
●	○	**PA4210**	***phzA1***	**probable phenazine biosynthesis protein**	**8.34**	**1.47**	**1788.72**	**1.26**	**6.27**	**1.49**	**13.02**	**2.11**	**63.05**	**1.33**
●	○	**PA4211**	***phzB1***	**probable phenazine biosynthesis protein**	**23.71**	**1.34**	**1256.93**	**1.20**	**4.25**	**1.25**	**39.82**	**2.16**	**50.65**	**1.21**
	○	PA4212	*phzC1*	phenazine biosynthesis protein PhzC			54.25	1.18			20.23	2.15	14.16	1.21
	○	PA4213	*phzD1*	phenazine biosynthesis protein PhzD			32.84	3.02			16.06	2.03	8.44	1.53
●	○	PA4214	*phzE1*	phenazine biosynthesis protein PhzE									6.89	1.23
	○	PA4215	*phzF1*	probable phenazine biosynthesis protein			208.87	1.25						
	○	PA4216	*phzG1*	probable pyridoxamine 5'-phosphate oxidase			25.83	1.35						
●		PA4217	*phzS*	flavin-containing monooxygenase			18.47	1.22						
●	○	PA4306	*flp*	Type IVb pilin, Flp			2.72	1.23						
		PA4348		conserved hypothetical protein			2.36	1.19						
		PA4384		hypothetical protein			2.60	1.16						
		PA4571		probable cytochrome c			2.25	1.27						
		PA4587	*ccpR*	cytochrome c551 peroxidase precursor			2.04	1.14						
		PA4649	*cupE2*	Pilin subunit CupE2			2.02	1.23						
		PA4916	*nrtR*	Nudix-related transcriptional regulator NrtR			2.36	1.21						
		PA5023		conserved hypothetical protein			2.07	1.11						
	○	PA5162	*rmlD*	dTDP-4-dehydrorhamnose reductase							2.16	1.11		
		PA5180		conserved hypothetical protein			4.96	1.16						
		PA5181		probable oxidoreductase			5.16	1.16						
		PA5219		hypothetical protein			2.40	1.15			2.17	1.14		
●		PA5220		hypothetical protein	2.70	1.10	15.23	1.16			6.49	1.23	3.33	1.11
		2870682957		hypothetical protein					2.57	1.08				
		2870698559		hypothetical protein									4.15	1.10
		2913673173		hypothetical protein			4.95	1.25					2.02	1.22
		2913675553		hypothetical protein	3.14	1.18	4.78	1.17			2.79	1.26		
		2913676290		ectoine hydroxylase-related dioxygenase (phytanoyl-CoA dioxygenase family)			2.54	1.16						
		2913679565		tRNA-dependent cyclodipeptide synthase			85.20	1.30						
		2913679566		hypothetical protein			8.44	1.34						
		2913679567		isopenicillin N synthase-like dioxygenase			4.86	1.28						
		2913679568		cytochrome P450			3.32	1.24						

2913679569	hypothetical protein			5.10	1.29						
2913679570	FIG00924305: hypothetical protein			3.82	1.29						
2913682544	hypothetical protein	5.43	1.13					13.25	1.24	2.38	1.18
2913682924	hypothetical protein	2.02	1.14								
2913689358	hypothetical protein							3.41	1.37		
2913690790	transcriptional regulator with XRE-family HTH domain							5.70	1.14		
2913690791	integrating conjugative element relaxase (TIGR03760 family)							2.28	1.15		
287.13131.peg.236	hypothetical protein			2.64	1.16						
287.13131.peg.2743	hypothetical protein			6.51	1.62						
287.13131.peg.2917	hypothetical protein			7.06	1.83						
287.13131.peg.4268	hypothetical protein			33.68	1.18						
287.13131.peg.6440	FIG00957537: hypothetical protein			2.49	1.31			8.64	1.45	3.58	1.30
287.13131.peg.793	FIG00958200: hypothetical protein			184.48	1.21	3.10	1.37				
287.13133.peg.4085	FIG00955856: hypothetical protein							7.14	1.27		
287.13133.peg.4120	hypothetical protein							5.98	1.39		
287.13133.peg.5885	hypothetical protein			5.29	1.25			4.34	1.30		
287.9771.peg.292	hypothetical protein					2.44	1.10				

[a]Locus tags, names, and functions from the PAO1 annotation on Pseudomonas.com (1), with the following exceptions: for genes not present in PAO1, the IMG gene ID and function are used (2, 3); for genes not present in PAO1 or IMG annotations, RAST gene ID and function are used (4); *aze* annotation from supplemental reference (5). ●, gene was reported as QS-regulated in PAO1 in supplemental reference (6). ○, gene shows evidence for direct transcriptional regulation by RhlR, or first gene in operon shows such evidence. **Bold** indicates genes in the core RhlR regulon reported in this study (Table 2, main text).

[b]Fold-change from individual comparisons between wild-type CF isolates and their isogenic *rhlR* mutants. FC, fold-change; FCSE, fold-change standard error.

References

1. Winsor GL, Griffiths EJ, Lo R, Dhillon BK, Shay JA, Brinkman FS. 2016. Enhanced annotations and features for comparing thousands of *Pseudomonas* genomes in the *Pseudomonas* genome database. *Nucleic Acids Res* 44:D646-53.
2. Chen I-MA, Chu K, Palaniappan K, Pillay M, Ratner A, Huang J, Huntemann M, Varghese N, White JR, Seshadri R, Smirnova T, Kirton E, Jungbluth SP, Woyke T, Eloe-Fadrosh EA, Ivanova NN, Kyrpides NC. 2018. IMG/M v.5.0: an integrated data management and comparative analysis system for microbial genomes and microbiomes. *Nucleic Acids Res* 47:D666-D677.
3. Mukherjee S, Stamatis D, Bertsch J, Ovchinnikova G, Sundaramurthi Jagadish C, Lee J, Kandimalla M, Chen I-MA, Kyrpides NC, Reddy TBK. 2020. Genomes OnLine Database (GOLD) v.8: overview and updates. *Nucleic Acids Res* 49:D723-D733.
4. Aziz RK, Bartels D, Best AA, DeJongh M, Disz T, Edwards RA, Formsma K, Gerdes S, Glass EM, Kubal M, Meyer F, Olsen GJ, Olson R, Osterman AL, Overbeek RA, McNeil LK, Paarmann D, Paczian T, Parrello B, Pusch GD, Reich C, Stevens R, Vassieva O, Vonstein V, Wilke A, Zagnitko O. 2008. The RAST Server: rapid annotations using subsystems technology. *BMC Genomics* 9:75.
5. Patteson JB, Lescallette AR, Li B. 2019. Discovery and biosynthesis of azabicyclene, a conserved nonribosomal peptide in *Pseudomonas aeruginosa*. *Organic Lett* 21:4955-4959.
6. Chugani S, Kim BS, Phattarasukol S, Brittnacher MJ, Choi SH, Harwood CS, Greenberg EP. 2012. Strain-dependent diversity in the *Pseudomonas aeruginosa* quorum-sensing regulon. *P Natl Acad Sci USA* 109:E2823-31.
