# Supplementary material for: Genetic and Transcriptomic Characteristics of RhlR-Dependent Quorum Sensing in Cystic Fibrosis Isolates of Pseudomonas aeruginosa
Source: mSystems. 2022 Apr 11;7(2):e00113-22. doi: 10.1128/msystems.00113-22 (PMC9040856; doi:10.1128/msystems.00113-22)
Supplement: FIG S2 [file msystems.00113-22-s0002.pdf]

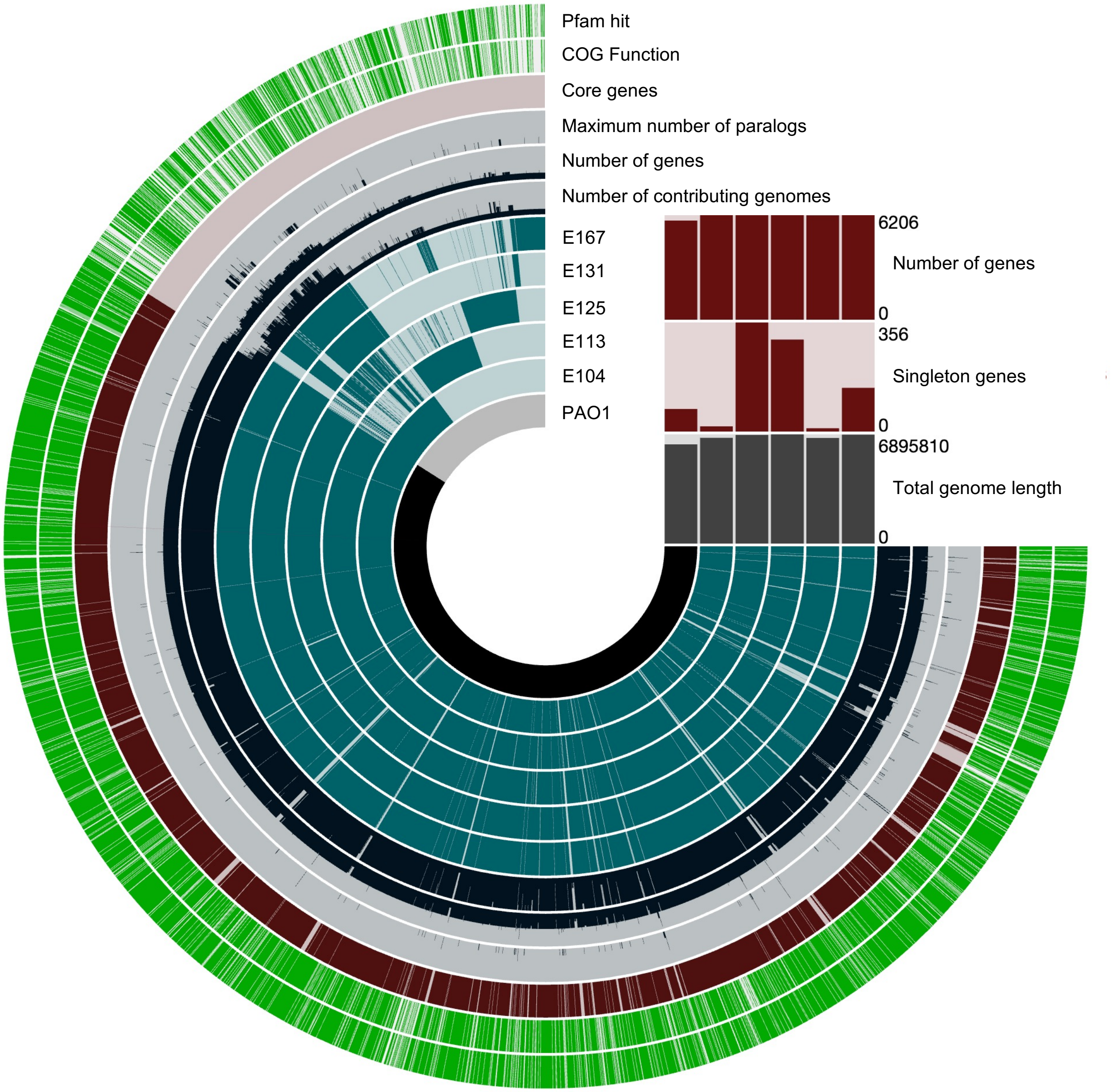

Figure S2. Pangenomic analysis. Analysis was conducted as described in the Materials and Methods. Loci are ordered by forced synteny to PAO1. Genes are indicated as presence/absence for the six inner tracks representing individual strains and the three outer tracks corresponding to Pfam hit or COG Function or Core genes. “Maximum number of paralogs”, “Number of genes”, and “Number of contributing genomes” are whole-number variables.
