# Supplementary material for: Genetic and Transcriptomic Characteristics of RhlR-Dependent Quorum Sensing in Cystic Fibrosis Isolates of Pseudomonas aeruginosa
Source: mSystems. 2022 Apr 11;7(2):e00113-22. doi: 10.1128/msystems.00113-22 (PMC9040856; doi:10.1128/msystems.00113-22)
Supplement: FIG S3 [file msystems.00113-22-s0003.pdf]

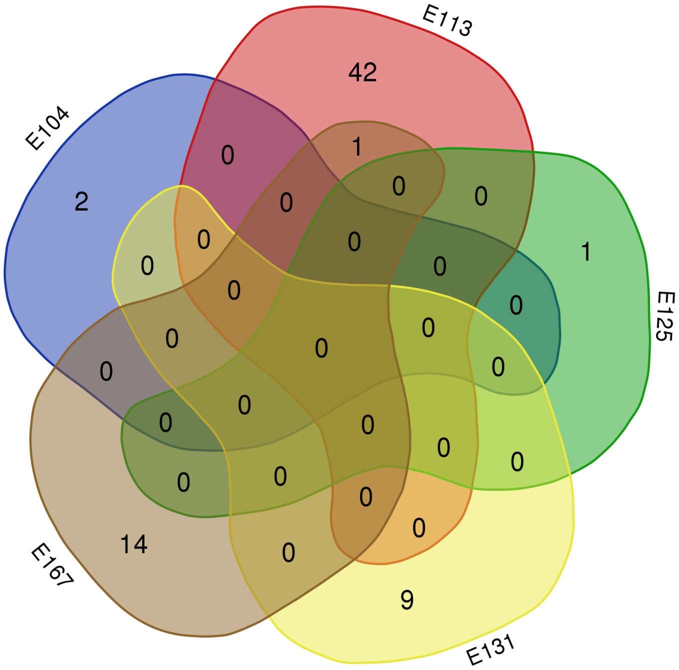

Figure S3. RhlR-repressed genes: core analysis. Venn analysis of genes identified as repressed in individual isolate differential expression analysis. Venn lobes are not scaled to regulon size.
