# Supplementary material for: Genetic and Transcriptomic Characteristics of RhlR-Dependent Quorum Sensing in Cystic Fibrosis Isolates of Pseudomonas aeruginosa
Source: mSystems. 2022 Apr 11;7(2):e00113-22. doi: 10.1128/msystems.00113-22 (PMC9040856; doi:10.1128/msystems.00113-22)
Supplement: TABLE S5 [file msystems.00113-22-s0008.pdf]

**Table S5. Bacterial strains and plasmids.**

| Strain or plasmid | Relevant properties | Reference or origin |
|---|---|---|
| *Pseudomonas aeruginosa* | | |
| PAO1 | Wild-type laboratory strain | (1) |
| PAO *lasR* | PAO1 derivative; "DA5" unmarked in-frame *lasR* deletion mutant | (2) |
| PAO *rhlR* | PAO1 derivative; "DA4" unmarked in-frame *rhlR* deletion mutant | (2) |
| PAO *lasR rhlR* | PAO1 derivative; "DA6" unmarked double-null deletion mutant in which both *lasR* and *rhlR* harbor in-frame deletions; '*lasR rhlR*' | (2) |
| E104 | Wild-type cystic fibrosis isolate | (3) |
| E104 *rhlR* | E104 derivative; unmarked in-frame *rhlR* deletion from residue 3-240 | This study |
| E113 | Wild-type cystic fibrosis isolate | (3) |
| E113 *rhlR* | E113 derivative; unmarked in-frame *rhlR* deletion from residue 3-240 | This study |
| E125 | Wild-type cystic fibrosis isolate | (3) |
| E125 *rhlR* | E125 derivative; unmarked in-frame *rhlR* deletion from residue 3-240 | This study |
| E131 | Wild-type cystic fibrosis isolate | (3) |
| E131 *rhlR* | E131 derivative; unmarked in-frame *rhlR* deletion from residue 3-240 | This study |
| E167 | Wild-type cystic fibrosis isolate | (3) |
| E167 *rhlR* | E167 derivative; unmarked in-frame *rhlR* deletion from residue 3-240 | This study |
| *Escherichia coli* | | |
| DH5α | F- Φ80lacZYA-argF U169 recA1 hsdR17 (rk-, mk+) phoA supE44 λ- thi-1 gyrA96 relA1 | Invitrogen |
| S17-1 | *recA* pro *hsdR* RP4-2Tc::Mu-Km::Tn7 | (4) |
| *Plasmids* | | |
| pEXG2 | Conjugative suicide plasmid for allelic exchange; $Gm^R$, *sacB* | (5) |
| pEXG2-E104rhlR-KO | pEXG2 containing an E104 strain-specific in-frame deletion allele of *rhlR* from amino acid 3-240 | This study |
| pEXG2-E113rhlR-KO | pEXG2 containing an E113 strain-specific in-frame deletion allele of *rhlR* from amino acid 3-240 | This study |
| pEXG2-E125rhlR-KO | pEXG2 containing an E125 strain-specific in-frame deletion allele of *rhlR* from amino acid 3-240 | This study |
| pEXG2-E131rhlR-KO | pEXG2 containing an E131 strain-specific in-frame deletion allele of *rhlR* from amino acid 3-240 | This study |
| pEXG2-E167rhlR-KO | pEXG2 containing an E167 strain-specific in-frame deletion allele of *rhlR* from amino acid 3-240 | This study |

| | | |
|---|---|---|
| pProbe-GT | Broad-host-range vector with a promoterless *gfp*, $Gm^R$ | (6) |
| pProbe-GT-$P_{lasI}$-*gfp* | pProbe-GT with *gfp* under the control of the *lasI* promoter | (3) |
| pBBR-*gfp* | Promoterless *gfp* transcriptional reporter, $Gm^R$ | (7) |
| pBBR-$P_{rhlA}$-*gfp* | pBBR-*gfp* with *gfp* under the control of the *rhlA* promoter | This study |

**References**


1. Stover CK, Pham XQ, Erwin AL, Mizoguchi SD, Warrener P, Hickey MJ, Brinkman FS, Hufnagle WO, Kowalik DJ, Lagrou M, Garber RL, Goltry L, Tolentino E, Westbrock-Wadman S, Yuan Y, Brody LL, Coulter SN, Folger KR, Kas A, Larbig K, Lim R, Smith K, Spencer D, Wong GK, Wu Z, Paulsen IT, Reizer J, Saier MH, Hancock RE, Lory S, Olson MV. 2000. Complete genome sequence of *Pseudomonas aeruginosa* PAO1, an opportunistic pathogen. *Nature* 406:959-64.
2. Siehnel R, Traxler B, An DD, Parsek MR, Schaefer AL, Singh PK. 2010. A unique regulator controls the activation threshold of quorum-regulated genes in *Pseudomonas aeruginosa*. *P Natl Acad Sci USA* 107:7916-7921.
3. Feltner JB, Wolter DJ, Pope CE, Groleau MC, Smalley NE, Greenberg EP, Mayer-Hamblett N, Burns J, Deziel E, Hoffman LR, Dandekar AA. 2016. LasR variant cystic fibrosis isolates reveal an adaptable quorum-sensing hierarchy in *Pseudomonas aeruginosa*. *mBio* 7 (5):e01513-16.
4. Simon R, Priefer V, Puhler A. 1983. A broad host range mobilisation system for in vivo genetic engineering: transposon mutagenesis in gram negative bacteria. *Biotechnol* 1:784-791.
5. Hmelo LR, Borlee BR, Almblad H, Love ME, Randall TE, Tseng BS, Lin C, Irie Y, Storek KM, Yang JJ, Siehnel RJ, Howell PL, Singh PK, Tolker-Nielsen T, Parsek MR, Schweizer HP, Harrison JJ. 2015. Precision-engineering the *Pseudomonas aeruginosa* genome with two-step allelic exchange. *Nat Protoc* 10:1820.
6. Miller WG, Leveau JHJ, Lindow SE. 2000. Improved *gfp* and *inaZ* broad-host-range promoter-probe vectors. *Mol Plant Microb In* 13:1243-1250.
7. Smalley NE, Schaefer AL, Asfahl KL, Perez C, Greenberg EP, Dandekar AA. 2022. Evolution of the quorum sensing regulon in cooperating populations of *Pseudomonas aeruginosa*. *mBio* 13:e00161-22.
